# Do patterns of past asbestos use and production reflect current geographic variations of cancer risk?: mesothelioma in Ontario and British Columbia, Canada

**DOI:** 10.1007/s10552-023-01672-4

**Published:** 2023-02-02

**Authors:** Catherine E. Slavik, Paul A. Demers, Lillian Tamburic, Hunter Warden, Christopher McLeod

**Affiliations:** 1 Occupational Cancer Research Centre, Ontario Health (Cancer Care Ontario), Toronto, ON M5G 1X3 Canada; 2 Dalla Lana School of Public Health, University of Toronto, Toronto, ON M5T 3M7 Canada; 3 Partnership for Work, Health and Safety, School of Population and Public Health, University of British Columbia, Vancouver, BC V6T 1Z3 Canada; 4 Faculty of Medicine, University of Toronto, Toronto, ON M5S 1A8 Canada

**Keywords:** Mesothelioma, Cancer incidence, Asbestos exposure, Spatial variation, Canada

## Abstract

**Purpose:**

Canada was a major global asbestos producer and consumer. Geographic patterns of Canadian asbestos use and mesothelioma, a highly fatal cancer linked to asbestos exposure, have not been previously reported. This study summarized key trends in mesothelioma incidence by geography and time in two Canadian provinces, Ontario and British Columbia (BC), and explored how past workforce characteristics and geographic trends in asbestos production and use may shape variations in regional rates of mesothelioma.

**Methods:**

We report trends in mesothelioma incidence (1993–2016) for Ontario and British Columbia using population-based incidence data that were age-standardized to the 2011 Canadian population. Historical records of asbestos production and use were analyzed to geo-locate industrial point sources of asbestos in Ontario and BC. The prevalence of occupations in regions with the highest and lowest rates of mesothelioma in Ontario and BC were calculated using labor force statistics from the 1981 Canadian Census.

**Results:**

Regional mesothelioma rates varied in both provinces over time; more census divisions in both Ontario and BC registered mesothelioma rates in the highest quintile of incidences during the period 2009 to 2016 than in any prior period examined. Certain occupations such as construction trades workers were more likely to be overrepresented in regions with high mesothelioma rates.

**Conclusion:**

This work explored how studying asbestos exposure and mesothelioma incidence at small-scale geographies could direct cancer surveillance and research to more targeted areas. Findings indicated that regional variations in mesothelioma could signal important differences in past occupational and potentially environmental exposures.

## Introduction

Asbestos has been described as a miracle mineral due to its unique properties for various industrial applications, most notably its durability and heat resistance [1]. As a result, asbestos was used in many industries for construction materials, insulation, brake materials, textiles and in transportation vehicles such as naval ships [2]. Canada played a large role in the industry and dominated global asbestos production throughout much of the 20th century while becoming a leading exporter [3]. Asbestos was mined in the Canadian provinces of British Columbia, Newfoundland and Labrador, Ontario and Quebec, as well as the Yukon Territory, with the largest volumes coming from Quebec [4]. Canada banned asbestos in 2018 [5] after the country’s last asbestos mine, the Jeffrey mine in Quebec, ceased operations in 2012 [6].

In addition to being a major asbestos producer and exporter, significant quantities of asbestos were also used and consumed domestically in Canada, primarily in the form of building materials such as cement products, floor tiles, ceiling tiles, roofing shingles, plaster and insulation, and in car parts [7]. For this reason, asbestos exposure outside of mining occurred, and continues to occur, most often among workers in the construction and transportation sectors (e.g., automotive repair, shipbuilding) [8]. These exposures pose a major hazard to workers because of the increased risk of cancer and lung disease [9]. Among these, mesothelioma, a cancer of the lining of the lungs and other internal organs, has become iconic for the impact of asbestos because it is almost exclusively linked to asbestos exposure. It is also well-tracked by tumour registries and its incidence has been increasing worldwide [10, 11]. Asbestos was first classified as a carcinogen by the International Agency for Research on Cancer in 1977 and although recognition of the deadly impacts of asbestos exposure began to grow globally [12], demand for asbestos did not start to decline for some time until the 1980s [3]. Its use in many lower income nations continues today [13].

Due to the long latency of cancer, that is the period of time between first exposure and cancer diagnosis, which can range from 10 to as long as 50 years [14], it is anticipated that mesothelioma incidence will continue to increase in countries like Canada that delayed implementing restrictions on its production and use [15]. Studies on asbestos consumption have already shown that countries with high usage consistently have higher rates of mortality from mesothelioma [16], but the risks vary considerably depending on the type of exposure. For example, the primary source of occupational exposure that has been linked to asbestos-related disease has shifted away from the mining sector to other industries such as construction, oil and gas and transportation repair [17, 18]. Still, with certain industries being associated with higher asbestos exposure relative to others, it is perhaps unsurprising that trends in mesothelioma incidence would emerge based on geographic differences in the labor force across regions [19, 20].

Yet, use of geographic information systems (GIS) have thus far been underutilized in occupational epidemiology [21], and only a few studies have applied a geographic lens to investigate occupational asbestos exposure and subsequent diagnoses of mesothelioma cases [22–24]. The goals of this study were to examine key trends in mesothelioma incidence by geography and time in two Canadian provinces and investigate whether past geographic trends in asbestos production and use could explain the variations in regional rates of mesothelioma. Our study focused on mesothelioma incidence rates between 1992 and 2018 due to constraints on cancer data availability prior to this period. However, due to the long latency of cancer, this study also collected data on asbestos production as far back as the 1960s to build a more complete history of potential exposures.

The two provinces examined in this work, Ontario and British Columbia, experienced peak asbestos production at different time periods in Canada and have different labor force characteristics making them an interesting set of cases to compare. Although the exposures that led to today’s cases of mesothelioma occurred decades ago, identifying which regions currently have the highest incidence rates and whether these regions share common asbestos exposure histories is useful in guiding present-day asbestos management policies and future healthcare system planning around mesothelioma care. Indeed, looking at where past asbestos exposures took place could offer important clues about where we might expect to see higher rates of mesothelioma in these provinces in the future and which workplaces and workers are most at-risk. The questions that will be explored in this research are:

- Q1: How have regional rates of mesothelioma in Ontario and British Columbia changed over time?
- Q2: Was asbestos used and/or produced in the regions with the highest mesothelioma rates in Ontario and British Columbia?
- Q3: Are certain occupations more prevalent in regions with the highest mesothelioma rates compared to regions with the lowest rates?
- Q4: How do rates of mesothelioma in Ontario and British Columbia compare to other Canadian provinces that produced asbestos?

## Methods

This study leveraged data from multiple sources and applied methods and analyses from epidemiology, geographic information science and occupational health. The current paper specifically sought to investigate the geography of mesothelioma and asbestos exposure in Ontario and British Columbia, as part of a larger study on the incidence and prognosis of mesothelioma in these two provinces. Consistent with Delaunay et al.’s [21] macro-approach to integrating industrial activity data with health-related data at regional scales, we also used maps to explore potential relationships between the location of asbestos-producing and asbestos-using industries and rates of mesothelioma by region. This approach broadly allows researchers to consider both the geographical and sectorial distribution of an industrial activity while simultaneously highlighting the distribution of work-related disease, which may be useful for guiding future policies and practices around health surveillance [21].

### Study areas

Ontario, located in central Canada, is the country’s most populous province and a significant manufacturing hub in the country [25]. Asbestos mines operated in Ontario between 1901 and 1977, primarily located in the northeastern part of the province with some small production from mines located in the southeast regions [26]. However, occupational asbestos exposure continued in the province into the 1980s due to the manufacturing of asbestos-containing products, e.g., automotive brakes and textile manufacturing [27]. Despite the present-day asbestos ban, occupational asbestos exposure continues today mostly through exposure to asbestos-containing materials during the repair, alteration, maintenance or demolition of older structures [8].

British Columbia is Canada’s most western province located along the Pacific coast. Only one asbestos mine was operational in British Columbia’s northwest region, the Cassiar Mine, which operated between 1942 and 1992 [28]. The province’s long coastlines feature a prominent shipbuilding industry, which has been linked to significant asbestos exposure and excess mesothelioma risk in other countries [29].

### Mesothelioma rates

To compare regional rates of mesothelioma (all sites) between 1993 and 2016 (in three 8-year periods), age-standardized incidence rates (ASIRs) of mesothelioma per 100,000 in Ontario were obtained from Cancer Care Ontario’s SEER*Stat program (version 8.3.8), with rates reported at a 95% confidence interval and standardized to the 2011 Census Canadian population (for males and females combined). Rates of mesothelioma per 100,000 residents (for males and females combined) from British Columbia were obtained from Population Data BC [30], an online secure research platform providing access to approved population and health administrative data in that province. These rates were also age-standardized to the 2011 Canadian population for the study period between 1993 and 2016 (in three 8-year periods). Rates from Ontario and British Columbia were aggregated by census divisions, which were classified and grouped by quintiles, and mapped using QGIS (version 2.14.3) [31]. Additionally, annual crude rates of mesothelioma by sex from 1992 to 2018 were obtained from the Canadian Cancer Registry (available online through Statistics Canada) [32] to compare rates from Ontario, British Columbia, and Quebec, another significant producer of asbestos in Canada.

### Geo-locating asbestos use and production

To geo-locate industrial point sources of asbestos use and production in Ontario and British Columbia, data on historical industrial locations associated with asbestos were collected from multiple publicly available datasets online. Data on the location of asbestos mines were obtained from historical records [26] and exact coordinates were obtained from Mindat, a web database on minerals and their locations around the world [33]. Additionally, geographic coordinates for four well-known asbestos processing plants in Ontario, which were operated by the H.W. Johns Manville Company during the late 1960s, were obtained using a Google search. Next, the geographic coordinates of industrial facilities that cited the use, storage or disposal of asbestos onsite in Ontario and British Columbia during any year between 1992 and 2006 were obtained from Canada’s National Pollutant Release Inventory [34] which corresponds to the approximate exposure window of asbestos and subsequent mesothelioma diagnoses, assuming a minimum latency of 10 years [14]. Finally, the locations of shipyards and boat manufacturing and repair facilities in Ontario and British Columbia that were operational between a relevant exposure period (between the 1960s-early 2000s) were obtained from a publicly maintained directory of Canadian shipbuilders [35]. The facilities were classified and grouped based on industry sector and mapped as point sources using QGIS.

To quantify asbestos production in Canada mined by province, Natural Resources Canada’s GEOSCAN tool [4] was used to obtain annual reports in Canadian Minerals Yearbooks annually from 1962 to 2009. Data on the annual production of asbestos in tonnes was linked with data on mesothelioma incidence rates from the Canadian Cancer Registry [32] by province and visualized using MS Excel.

### Linking occupations to cancer incidence

To compare the prevalence of occupations in regions with the highest and lowest rates of mesothelioma in Ontario and British Columbia, labor force statistics were obtained from the 1981 Canadian Census [36]. The 1981 Census was selected due to the minimum latency of 10 years for mesothelioma after exposure to asbestos. Census publications prior to this period did not publish detailed labor force statistics by both minor occupational groups and census divisions. Census divisions in the highest and lowest quintile of mesothelioma incidence rates over the whole study period (between 1993 and 2016) were identified in Ontario and British Columbia and the number of workers (aged 15 years and over) employed in each major occupation group was collected. From this dataset, the proportion of the total workforce in each census division that was employed in each occupational group was calculated.

To compare the proportions of workers employed per occupation in high versus low mesothelioma incidence quintile groupings, the proportion was averaged across census divisions in the high and low incidence groups in Ontario and in British Columbia. Two-sided proportion tests at a 95% confidence interval were carried out in the R statistical computing software [37] to examine whether the proportions among census divisions in the high versus low quintiles differed for every occupation. The proportions were transformed into percentages by multiplying by 100 and visualized using MS Excel.

## Results

### Changes in mesothelioma incidence rates over time

**Fig. 1:**
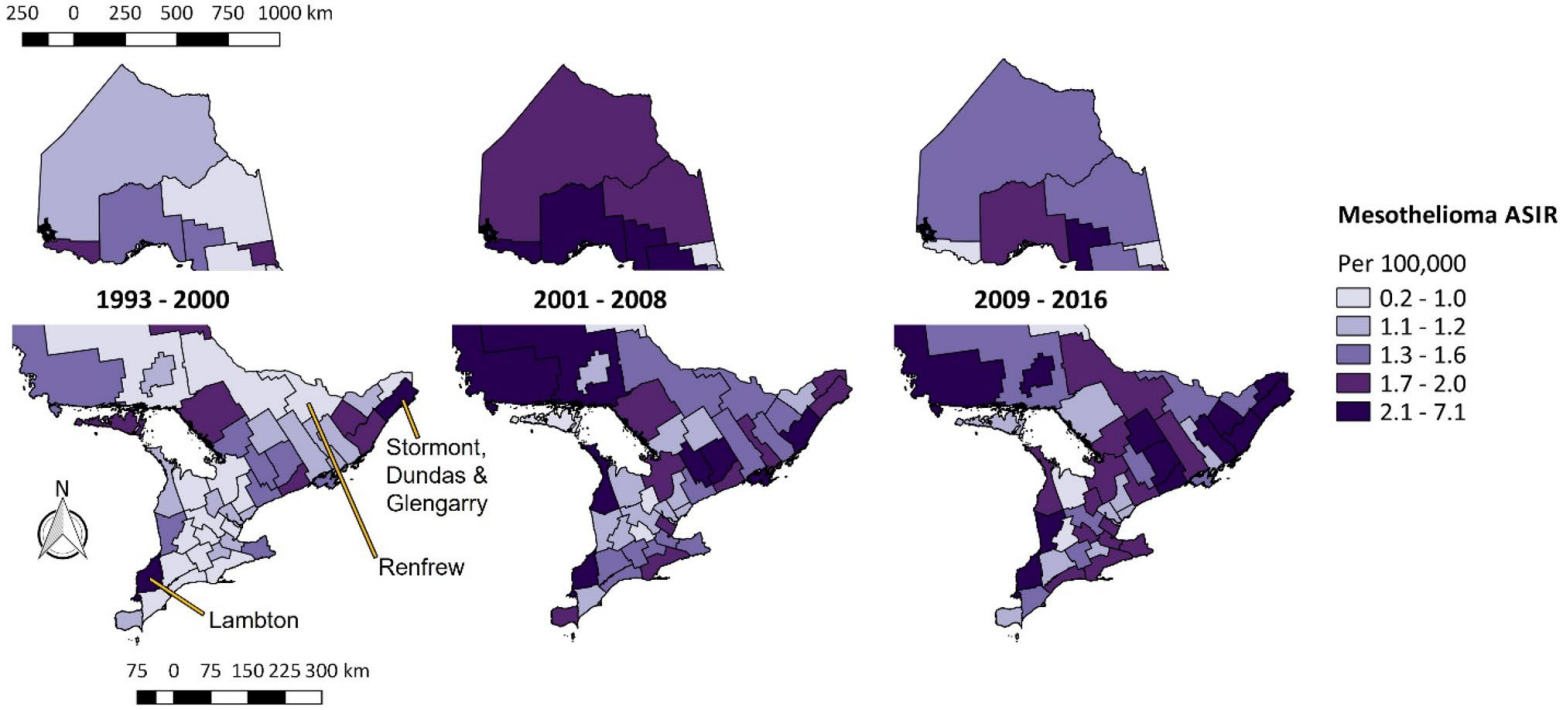
Map of age-standardized incidence rates of mesothelioma in Ontario over time, 1993–2016

In Ontario, the incidence rates of mesothelioma changed over time (Fig. 1). In the period between 1993 and 2000, only two census divisions, Lambton in the southwest of the province and Stormont, Dundas and Glengarry on the eastern-most edge of Ontario, had mesothelioma rates in the highest quintile, with rates of 4.7 per 100,000 and 2.4 per 100,000, respectively. By the period 2001 to 2008, census divisions in northern Ontario saw noticeable changes in incidence rates from lower to higher quintiles. In all, 10 census divisions were in the highest quintile of mesothelioma rates between 2001 and 2008, with Lambton recording the highest rate of 7.1 per 100,000. Between 2009 and 2016, higher mesothelioma incidence rates were obeserve in more regions in eastern Ontario, bringing the total number of census divisions with mesothelioma rates in the highest quintile to 12. During this period, the census division with the highest incidence rate of mesothelioma remained Lambton at 4.4 per 100,000. The census division with the largest percentage change in mesothelioma incidence rates over the whole study period was Renfrew, located west of the national capital Ottawa, which saw its rates go from 0.2 per 100,000 during the period 1993–2000 to 1.5 per 100,000 during the period 2009–2016, corresponding to a greater than 500% increase.

**Fig. 2:**
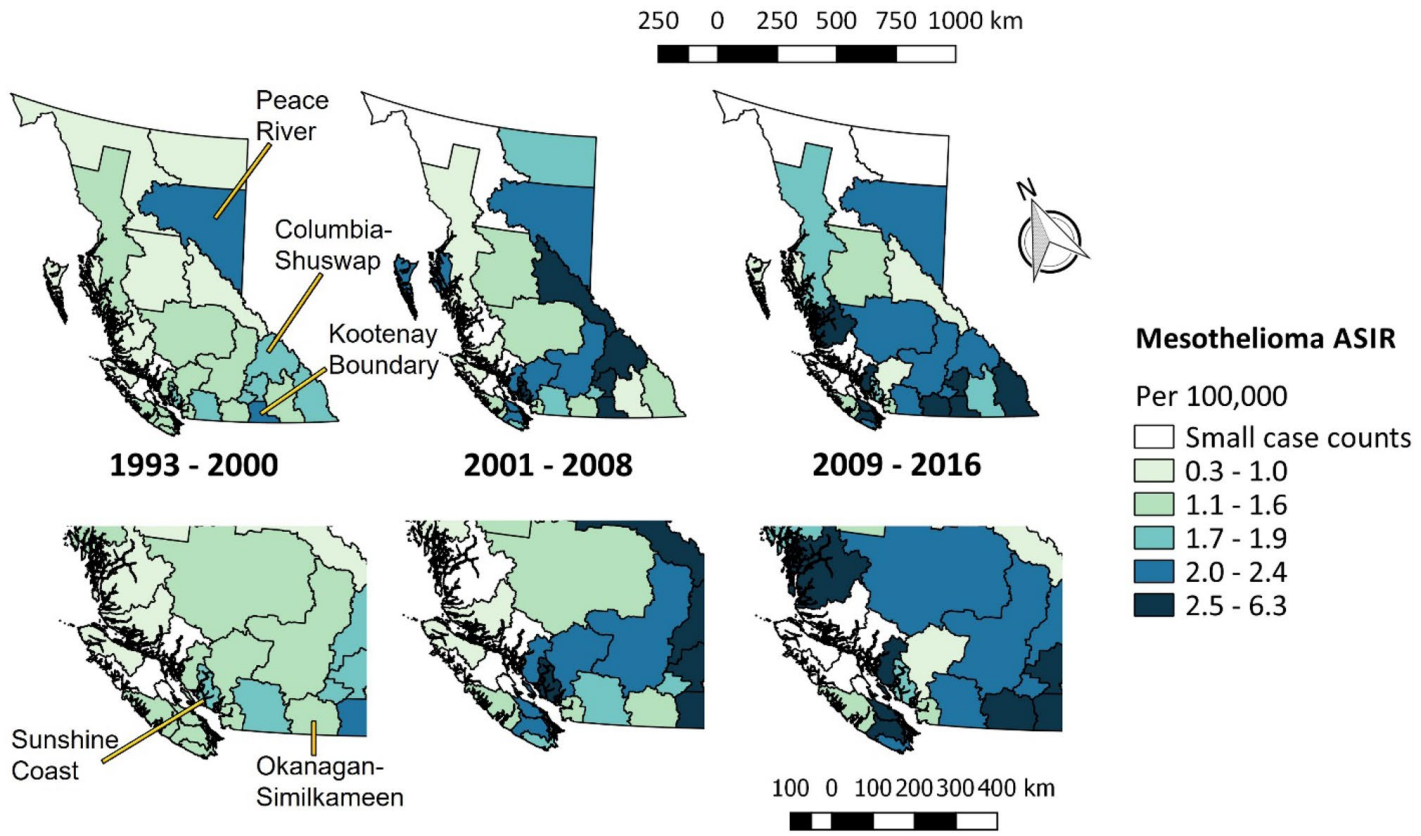
Map of age-standardized incidence rates of mesothelioma in British Columbia over time, 1993–2016

In British Columbia, incidence rates for mesothelioma have also undergone regional changes over time (Fig. 2). Between 1993 and 2000, the highest incidence rates were recorded in Kootenay Boundary on the southern edge of the province bordering the United States, and in Peace River in the northeast of the province, with rates of 2.4 per 100,000 and 2.2 per 100,000, respectively. During the period between 2001 and 2008, 5 census divisions in British Columbia saw mesothelioma incidence rates in the highest quintile. The census division with the highest rate of mesothelioma during this time was still Kootenay Boundary (3.4 per 100,000), followed by Columbia-Shuswap in the province’s interior and Sunshine Coast just north of Vancouver, both with rates at 3.2 per 100,000. Between 2009 and 2016, 9 census divisions had incidence rates of mesothelioma in the highest quintile, with the highest rate occurring again in Kootenay Boundary (6.3 per 100,000). The census division with the largest percentage change in mesothelioma incidence rates over the whole study period was Okanagan-Similkameen, located just west of Kootenay Boundary along the province’s southern border with the United States, which saw its rates go from 1.4 per 100,000 during the period 1993–2000 to 4.1 per 100,000 during the period 2009–2016, an increase of approximately 200%.

### Asbestos use and production in Ontario and British Columbia

**Fig. 3:**
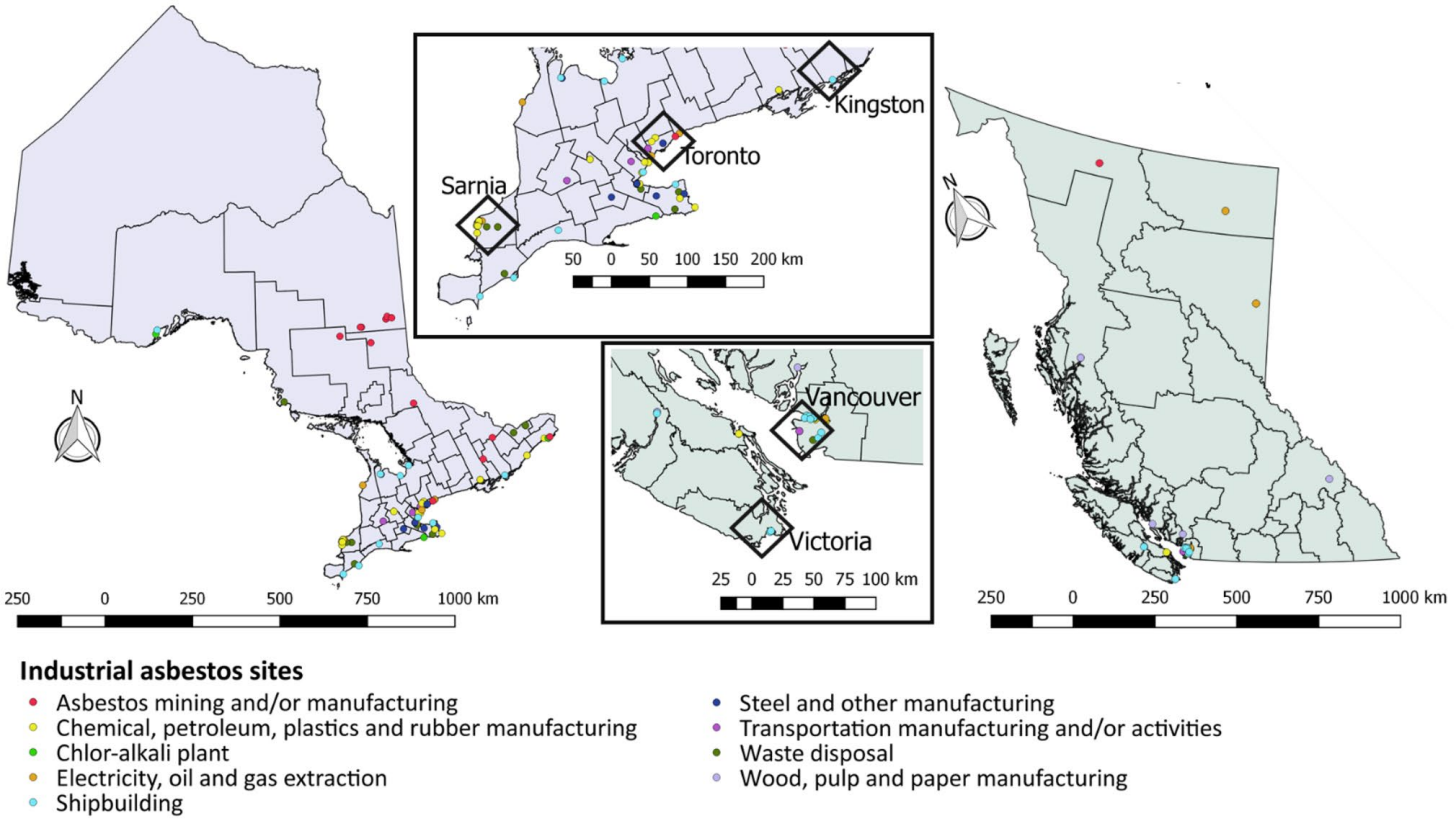
Map of industrial sites with historical use and/or production of asbestos in Ontario and British Columbi

To examine locations with historical asbestos use and production in Ontario and British Columbia, relevant point sources and industrial facilities are mapped in Fig. 3. In Ontario, facilities were distributed across the southern part of the province, with some industries clustered in particular areas. For example, around the City of Sarnia (Fig. 3), which is located in Lambton (i.e., the census division consistently with the highest incidence rates of mesothelioma in Ontario) (Fig. 2), there was a clustering of facilities in the chemical, petroleum, plastics and rubber manufacturing industries. In and around the City of Toronto (Fig. 3), a considerable variety of industries associated with historical asbestos use and processing existed and continue to exist. Numerous shipbuilding industries also existed in ports along the edges of the Great Lakes, with some still in operation today.

In British Columbia, the majority of industrial facilities with historical asbestos use and production were located around the cities Vancouver and Victoria. In these locations, there were (and continue to be) numerous shipyards. Other notable facilities included the wood, pulp and paper manufacturing industries located along the Pacific coast of the province and within its interior region.

### Comparison of workforce in regions with the highest and lowest rates of mesothelioma

**Fig. 4:**
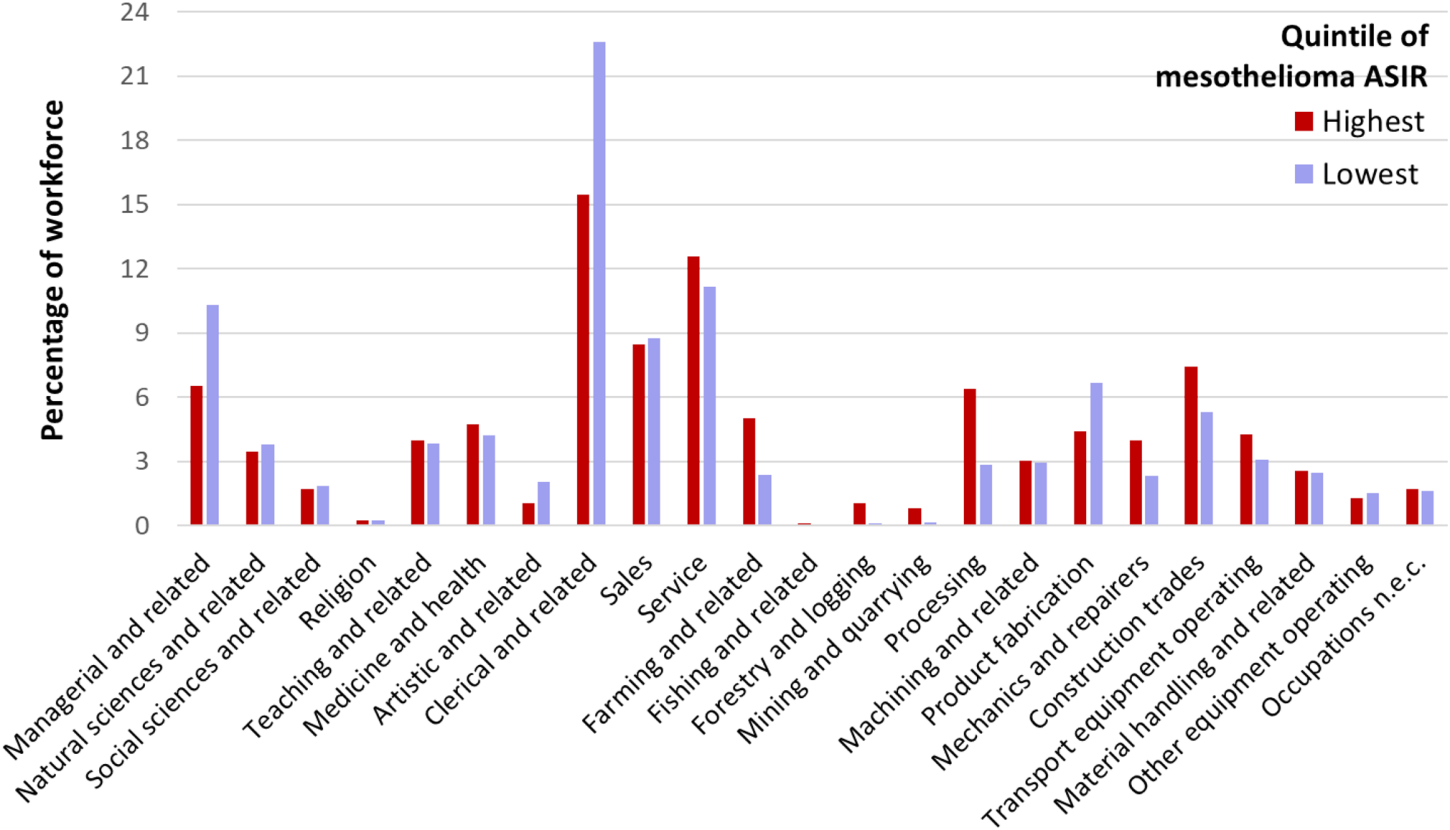
Mean percentage of total workforce employed (in 1981) in each occupation group, by census divisions in the highest quintile of age-standardized incidence rates (ASIR) versus lowest quintile of ASIR for mesothelioma, Ontario, 1993–2016

When comparing proportions of the workforce employed in census divisions with the highest incidence rates of mesothelioma versus the lowest in Ontario, some differences were observed by occupation from 1981 (Fig. 4). The largest difference was among clerical and related occupations; census divisions with the lowest rates of mesothelioma (between 1993 and 2016) on average had 22.6% of their workforce employed in this occupation in 1981 compared to 15.4% of the workforce employed in census divisions with the highest rates of mesothelioma (*p* < 0.001). We observed a similar trend among managerial and related occupations in Ontario, where census divisions with the lowest rates of mesothelioma on average had 10.3% of their workforce employed in this occupation compared to 6.5% in census divisions with the highest rates of mesothelioma (*p* < 0.001).

Census divisions in Ontario with the highest incidence rates of mesothelioma (between 1993 and 2016) had significantly higher percentages of their workforce employed in occupations related to material processing (6.4%), farming (5.0%) and construction (7.4%) compared to census divisions with the lowest rates (2.8%, 2.4%, 5.3%, respectively) in 1981. The differences in these proportions were all statistically significant (*p* < 0.001). Percentages were also higher in census divisions with the highest mesothelioma incidence rates compared to census divisions with the lowest rates for occupations related to service (12.6% vs. 11.2%), forestry (1.0% vs. 0.1%), mining (0.8% vs 0.1%), mechanics and repairers (3.9% vs. 2.3%) and in transport equipment operating (4.3% vs. 3.0%). Although the absolute differences in proportions were smaller for these occupations between the highest and lowest quintiles, they were all statistically significant (*p* < 0.001).

**Fig. 5:**
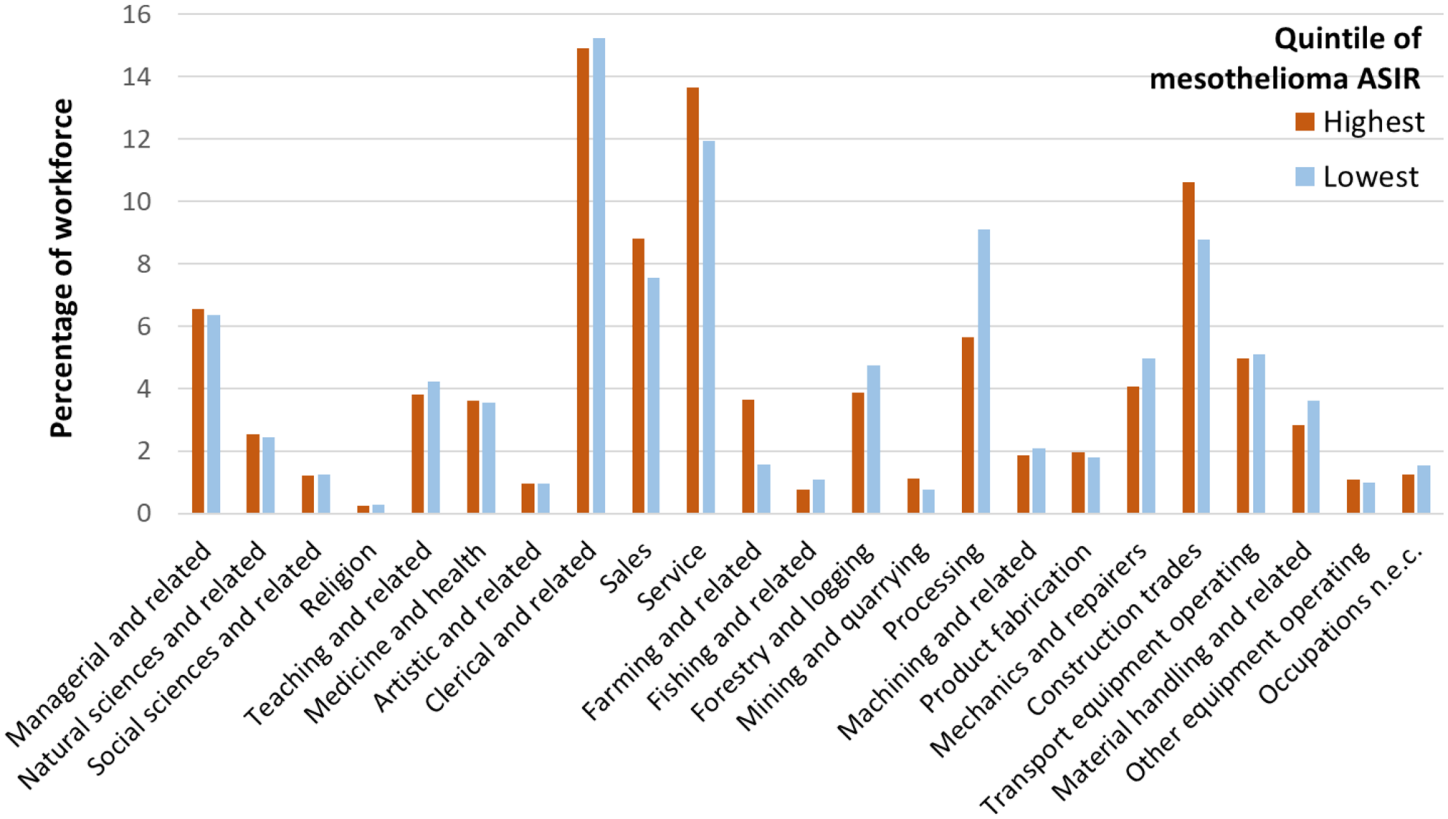
Mean percentage of total workforce employed (in 1981) in each occupation group, by census divisions in the highest quintile of age-standardized incidence rates (ASIR) versus lowest quintile of ASIR for mesothelioma, British Columbia, 1993–2016

When comparing proportions of the workforce employed in 1981 in census divisions with the highest incidence rates of mesothelioma versus the lowest rates in British Columbia (1993–2016), some key differences were observed by occupation (Fig. 5). The largest difference occurred in processing occupations, where the percentage of the workforce employed was significantly higher in census divisions in the lowest mesothelioma incidence quintile (9.1%) compared to census divisions in the highest mesothelioma incidence quintile (5.5%) (*p* < 0.001). This relationship was also seen for occupations in forestry and logging, in which 4.7% of the workforce was employed in census divisions in the lowest quintile versus 3.9% in the highest quintile (*p* < 0.001), and mechanics and repairers (5.0% vs. 4.0%, *p* < 0.001). The reverse trend was observed for these three occupations in Ontario, with higher proportions of the workforce employed in these occupations in census divisions in the highest ASIR quintile for mesothelioma compared to the lowest quintile, as reported previously.

On the other hand, census divisions in British Columbia in the highest quintile of mesothelioma incidence rates employed larger percentages of their workforce in occupations-related farming (3.6%), construction (10.6%) and mining (1.1%) relative to census divisions in the lowest quintile of mesothelioma incidence (1.6%, 8.8%, and 0.8%, respectively) (*p* < 0.001). These trends in occupations were consistent with those observed in Ontario. Percentage of workers employed in sales and service occupations were also higher in the highest quintiles of mesothelioma (8.8% and 13.7%, respectively) compared to the lowest quintiles (7.6% and 11.9%, respectively), which was a trend only observed for service occupations in Ontario.

### Mesothelioma in other Canadian provinces with asbestos mining

**Fig. 6:**
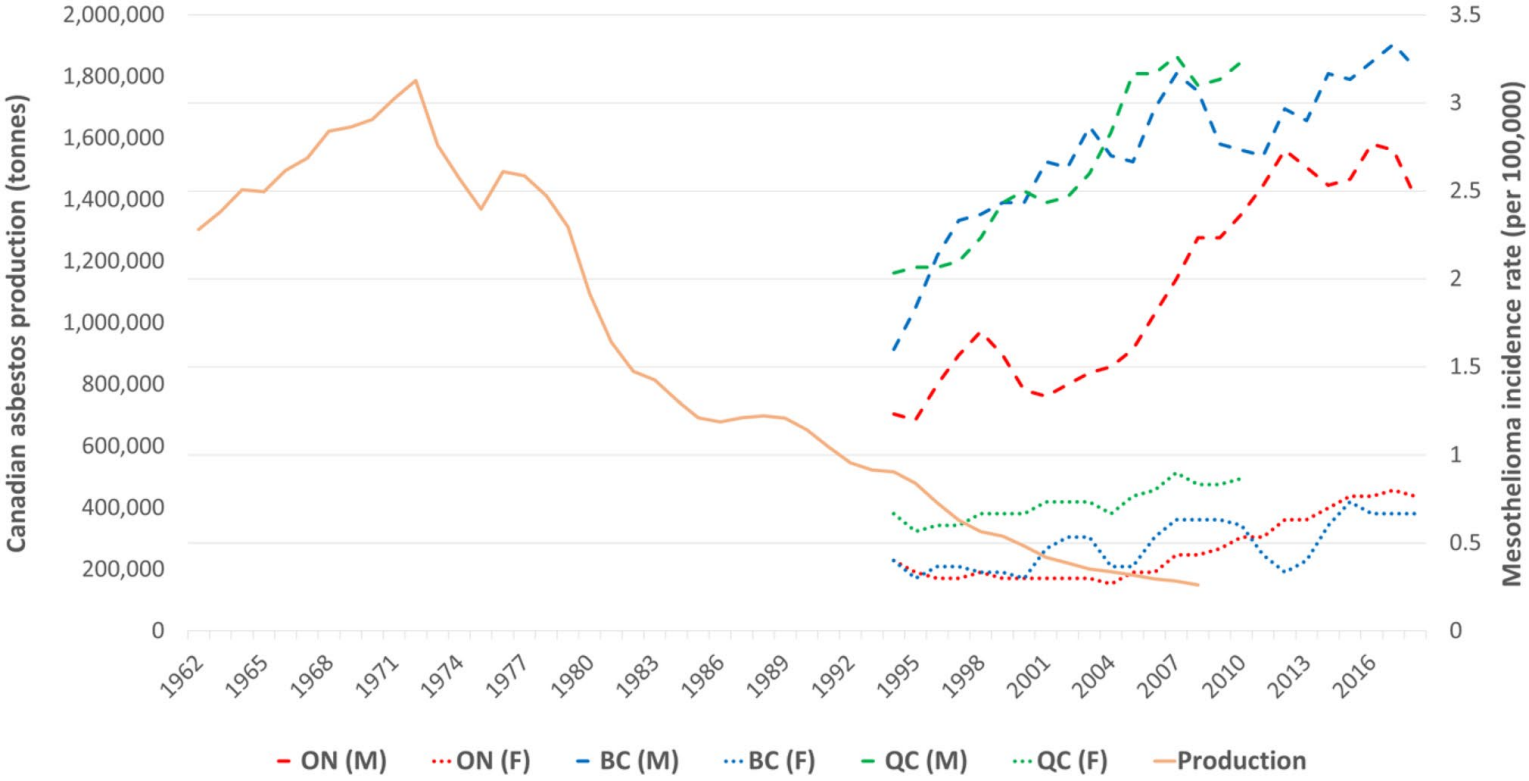
Asbestos production in Canada (1962–2009) and incidence rates of mesothelioma, by sex and select province (1992–2018), using three-year rolling average. Note: Quebec (QC) cancer data were unavailable after 2010 from the Canadian Cancer Registry. (M) = Male rates. (F) = Female rates. Using Bonferroni-corrected two-way analysis of variance, differences in rates among males were significant between Ontario and British Columbia (*p* < .0001) and between Ontario and Quebec (*p* < .0001) but not between British Columbia and Quebec (*p* = 0.424). Differences in rates among females were significant between Quebec and Ontario (*p* < .0001) and between Quebec and British Columbia (*p* < .0001) but not between Ontario and British Columbia (*p* = 0.999), using the same tests as for males

Asbestos production peaked in Canada in the early 1970s (Fig. 6), which mainly took place in the Province of Quebec. Of the total asbestos produced, the lowest volumes came from mining in Ontario (approximately 400 tonnes, ending in 1977), with much larger volumes mined in British Columbia (approximately 2500 tonnes, ending in 1992) and Quebec (approximately 37,300 tonnes, ending in 2010) (data not shown). The trends in mesothelioma incidence over time by province were consistent with the volumes of asbestos mined in each province, with rates appearing to be highest in Quebec, followed by British Columbia and Ontario, although rates among females in Ontario have surpassed those of females in British Columbia in recent years. Unfortunately, incidence rates for Quebec are not available after 2010 to compare the rates of mesothelioma across the three provinces during the last decade.

## Discussion

This study used a geographical approach to investigate current trends in mesothelioma incidence in two Canadian provinces, Ontario and British Columbia, and examined variations in potential sources of past asbestos exposure. We found a changing landscape of mesothelioma diagnoses across both provinces, with more regions facing higher rates of the cancer in the period 2009–2016 than in previous periods examined. While some regions maintained consistently high rates (e.g., Lambton in Ontario and Kootenay Boundary in British Columbia), other regions gradually saw their rates of mesothelioma increase or decrease over time. In Ontario, the shifting burden of the disease from the north to the eastern regions of the province are consistent with timelines of peak asbestos exposure by region; asbestos mining ended in the northern regions in the late 1970s, however, asbestos continued to be used in various manufactured products and industrial processes in other regions of the province. In Kootenay Boundary, British Columbia, significant metal smelting and refining operations existed and continue to exist, which were known to use asbestos for fireproofing and could partly explain the high rates of mesothelioma there.

Our study also points to the potential value of provincial asbestos registries and more detailed exposure histories to guide mesothelioma surveillance and management. Although our investigation found numerous possible point sources where asbestos was used and produced in Ontario and British Columbia, our list of locations is surely an underestimation of workplaces and buildings where asbestos exposure has occurred. This is because many of the sources of data we used to build our dataset of potential asbestos exposure were created after peak use and records of historical use are not always available. The locations we found in Ontario and British Columbia where asbestos was used and produced (Fig. 3) did line up with some regions that have high rates of mesothelioma (e.g., Lambton, Ontario and southern regions of Vancouver Island, British Columbia). However, our study would also suggest that more cases of mesothelioma should be occurring around the Greater Toronto Area and City of Vancouver, the absence of which indicates that other factors –such as a small overall proportion of occupationally exposed urban populations or the movement of people from cities to other regions– could be contributing to higher rates of mesothelioma diagnosed in other places.

Given that numerous published studies have found elevated risks of mesothelioma among workers in certain industries and occupations [38–40], we expected to see a high proportion of workers in industries known to carry higher risks of asbestos exposure employed in regions with higher mesothelioma rates and a lower proportion of workers in other industries employed in regions with lower mesothelioma rates. For example, we anticipated that occupations related to clerical and managerial work would be associated with less asbestos exposure and therefore a smaller proportion of the workforce would be employed in clerical and managerial occupations in regions with the lowest mesothelioma rates, which was observed in Ontario (Fig. 4). However, in British Columbia, the proportion of workers with these occupations was similar in regions with the highest and lowest rates of mesothelioma (Fig. 5), which suggests that worker characteristics and/or workplace conditions for this occupational group did not significantly differ across regions with differing rates of mesothelioma.

The occupations where we expected to see workers overrepresented in regions with high mesothelioma rates were in the construction trades, mining, processing, and mechanics and repairers consistent with previous research [18]. Processing is a broad occupational group including workers involved with the processing of materials such as metals, chemicals, petroleum, rubber, plastic, wood and pulp, mineral ore treatment, and textile processing. This trend was observed in Ontario (Fig. 4), but in British Columbia, the percentage of workers in occupations related to processing and mechanics and repairers was higher in regions with lower rates of mesothelioma compared to regions with higher incidences of mesothelioma. This was a surprising finding, which points to a potential disconnect between historical workforce characteristics (i.e., historical exposures) and current-day cancer risk in British Columbia. This observed disconnect could be attributed to differences in the levels of asbestos exposure occurring among select occupations in British Columbia compared to Ontario, or perhaps due to differences in other factors such as people’s migration within and out of the province.

Another surprising finding was the higher percentage of people working in occupations related to farming in regions with the highest mesothelioma rates compared to regions with the lowest mesothelioma rates in both Ontario and British Columbia. Most epidemiologic studies have not revealed higher risks of mesothelioma among agricultural workers [41], though opportunities for asbestos exposure have been identified in this sector [42]. While this study did not quantify actual asbestos exposures among farming occupations to examine whether this result represents a true increased risk of mesothelioma among workers in this occupation group, it does help to illustrate why examining trends in worker exposures and disease at small-scale geographies can be useful to test new hypotheses surrounding who is at-risk. In the absence of detailed occupational exposure histories collected from individuals with cancer, we have shown that comparing the prevalence of occupations in areas with high versus low mesothelioma incidence rates can still be a useful approach to help set future regional priorities for disease surveillance in certain occupations and industries.

Despite asbestos mining having ended in both Ontario and British Columbia decades ago, the varying geographic patterns of mesothelioma diagnoses across both provinces that we have presented suggest that the industrial use of asbestos (rather than production) was more important at driving regional disease trends. In the province of Quebec, where nearly 10 times as much asbestos was mined until as recently as 2010, the geographic patterns of mesothelioma diagnoses may end up looking very different from those in Ontario and British Columbia and may be driven by trends in mining to a larger extent. Thus, a geospatial analysis of mesothelioma in Quebec would make for an interesting follow-up to this work and may reveal higher rates of the disease in regions where mining occurred. However, due to the long latency of cancer and the relatively recent end to asbestos production in Quebec, trends in mesothelioma incidence may continue take some time to develop there. In fact, it has been suggested that low risk perceptions regarding exposure to asbestos fibres, coupled with asbestos mining residues covering significant areas, will contribute to ongoing mesothelioma cases for decades to come in southeastern Quebec [43]. Therefore, while rates of mesothelioma appear to be plateauing or declining in some regions of Ontario and British Columbia, cases may continue to rise in other regions of Canada such as Quebec.

A major strength of this work was leveraging publicly available data to create a geo-coded dataset of potential past and present asbestos exposure sites in Ontario and British Columbia. Since a comprehensive database of asbestos in workplaces and in buildings does not exist in either province (only a national inventory of asbestos in buildings owned by the Canadian federal government and a registry of asbestos in public buildings in the province of Saskatchewan are currently available), this study collected data on the locations of numerous facilities with asbestos present to explore geographic patterns in exposure and disease, though it’s possible that key workplaces in many regions were missed. Still, our study does highlight some occupations that may require more surveillance for asbestos-related diseases, which could be helpful to governments in regions where those occupations are common.

Our study has some important limitations that are important to consider. First, although occupational exposures to asbestos have been the leading cause of mesothelioma for many years, as restrictions and bans on asbestos use and production have come onboard in many places, it is likely that environmental and neighbourhood exposures will become increasingly important to study [44]. For example, the residential proximity to asbestos deposits has been found to be an important geographic variable associated with increased risk of mesothelioma [45], which would make for an interesting future study expanding on the work we have done. Second, we presented incidence rates regionally by combining cases from both sexes in Ontario and British Columbia due to small numbers; however, since males make up significantly more cases overall, analyzing occupations from the Census from males only could have provided a more accurate picture of the percentage of the workforce in key occupations contributing to cases of mesothelioma in each region. That being said, combining data from both males and females likely resulted in a more conservative analysis. Still, other studies have pointed to incidences of mesothelioma among females (who often lack a documented occupational asbestos exposure) as an indicator of the importance of environmental asbestos exposure to mesothelioma diagnoses [22, 46], which would make for an interesting next area of study in a follow-up to this work.

## Conclusion

This work has implications for health researchers and government officials working at various scales looking to identify patterns in hazardous exposures and disease incidence. Although restrictions and bans on asbestos use have led to declines in mesothelioma in many places [47], the continued use of asbestos elsewhere means there are still opportunities to implement policies that limit exposure. As Canada played a key role in supporting asbestos markets worldwide [48], it has many lessons to share with other nations now seeing significant increases in cases of mesothelioma.

One key lesson, exemplified through this work, is the importance of studying trends in mesothelioma at small-scale geographies due to local variations in sources of asbestos exposure from varying industry sectors and processes. Our study has illustrated that regional variations in mesothelioma could be a sign of important differences in past occupational and potentially environmental exposures. Studying these differences in past occupations, as well as gender-based differences in exposures, could lead to more precise models of future mesothelioma cases informed by local labor characteristics and could help direct asbestos-related disease surveillance going forward.

Lastly, this work points to the ongoing need to establish registries of industrial facilities and buildings that contained asbestos, which would allow researchers to track exposures over time and identify individuals at greatest risk of mesothelioma and other asbestos-related disease. Despite bans on asbestos, this work has highlighted the ongoing need to conduct surveillance of asbestos exposure and mesothelioma incidence rates in many places in Canada and beyond, where rates of mesothelioma are likely still increasing.

## Data Availability

The dataset containing locations of historic asbestos industrial sites generated for this project are available upon request. Mesothelioma cancer incidence data by small-scale geography were obtained from provincial cancer registries are not publicly available due to the small numbers of cases in some regions. Other datasets used in this work are already publicly available online.
